# Exploring Determinants of Adherence to the Mediterranean Diet Among Adults in Lebanon During the Economic Crisis: A Qualitative Study

**DOI:** 10.1111/jhn.70053

**Published:** 2025-04-15

**Authors:** Cécile Obeid, Anke Oenema, Antoine Aoun, Luna Awad, Stef P. J. Kremers, Jessica S. Gubbels

**Affiliations:** ^1^ Department of Health Promotion, NUTRIM Institute of Nutrition and Translational Research in Metabolism Maastricht University Maastricht the Netherlands; ^2^ Department of Health Psychology Open University Heerlen the Netherlands; ^3^ Faculty of Nursing and Health Sciences Notre Dame University Zouk Mosbeh Lebanon; ^4^ Center for Obesity Prevention Treatment Education and Research (COPTER) Notre Dame University‐Louaize Zouk Mosbeh Lebanon; ^5^ Department of Counseling and Health Notre Dame University‐Louaize Zouk Mosbeh Lebanon

**Keywords:** adherence, determinants, economic crisis, Mediterranean diet, qualitative study, socio‐ecological model

## Abstract

**Introduction:**

Adherence to MD is declining in its native region, posing public health concerns. To improve adherence, it is important to understand its determinants. Deriving from socio‐ecological models (SEM), determinants may include socio‐demographic, environmental and behavioural factors. Lebanon is a Mediterranean country that faces serious challenges due to an ongoing economic crisis, which may affect MD adherence and its determinants. The aim of this study is, therefore, to explore the determinants influencing adherence to the MD in Lebanon, as well as the impact of the economic crisis on these factors.

**Methods:**

This study employed a qualitative approach consisting of individual face‐to‐face interviews. A purposive sample of 25 generally healthy adults from different regions in Lebanon was recruited. Data collection started with a brief questionnaire to collect background socio‐demographic information, followed by assessing MD adherence using the Mediterranean Diet Adherence Screener. Next, participants were interviewed using a semi‐structured interview guide, focusing on barriers and enablers to adhering to the MD.

**Results:**

Participants maintained a moderate adherence to MD with many reporting improving or sustaining healthy eating habits. Because of the economic crisis, the consumption of local produce became more prevalent due to import shortages, aligning with MD recommendations. Determinants included individual attitudes beliefs, particularly concerns about the quality and trustworthiness of food products during the economic crisis, and environmental factors like the unavailability of imported foods. Convenience and proximity were prioritized over cost in food purchases.

**Conclusion:**

Despite economic constraints, many participants prioritized diet quality. People shifted to traditional, healthy diets. Emphasizing tradition, food availability, accessibility and affordability could be crucial for interventions.

## Summary

- Adherence to the Mediterranean Diet (MD) in Lebanon remains moderate, with many individuals maintaining or improving their eating habits despite economic challenges.
- The ongoing economic crisis has influenced food choices, leading to an increased reliance on locally produced foods due to import shortages, aligning with MD recommendations.
- Determinants of MD adherence include individual attitudes and beliefs, particularly concerns about food quality and trustworthiness amid the crisis.
- Environmental factors such as food availability and convenience play a crucial role, with proximity being prioritized over cost in food purchasing decisions.
- Promoting traditional diets and emphasizing food accessibility and affordability may enhance adherence to the MD, highlighting key areas for intervention.

## 1 Introduction

The Mediterranean diet (MD), based on generous consumption of olive oil, fish, legumes, fruits and vegetables with limited intake of red meat and processed food [1], has long been praised for its health benefits, such as protection against cardiovascular diseases and Alzheimer's disease [2]. However, despite these health benefits, adherence to the MD is declining in the Mediterranean region. A systematic review showed that adherence to the MD among inhabitants of its countries of origin (i.e., the Mediterranean region) was low to moderate during the past 10 years [3]. Also, Lebanon, a Middle Eastern country on the eastern shore of the Mediterranean Sea, has traditionally adhered to the MD [4]. However, the few previous studies from Lebanon specifically, indicate a decline in MD adherence, with reports of reduced consumption of key components such as bread, fruits, vegetables, legumes, milk, eggs and olive oil, alongside increased intake of processed foods, sugary beverages, red meats, added fats and oils, and cereal‐based products [5]. A study comparing dietary trends between 1997 and 2008/2009 found significant decreases in nutrient‐dense foods like fruits, while energy‐dense foods, such as chips, crackers and sweetened beverages saw an increase [6]. A 2018 study further confirmed declining MD adherence scores, highlighting a continued shift away from traditional dietary patterns towards less nutrient‐dense, more energy‐dense foods [5]. The decline in adherence to the MD in Mediterranean countries in general and in Lebanon more specifically raises red flags for public health experts and researchers alike, underscoring the importance of promoting re‐adoption and continuation of following the MD in these countries. To do so, it is important to understand the determinants of MD adherence at various levels [3]. While the benefits of the MD are widely acknowledged [7], the underlying determinants influencing individuals' decisions to adhere or deviate from this dietary pattern remain inadequately explored particularly in the non‐European Mediterranean countries [3], especially when examined through the lens of socio‐ecological models (SEMs) [8]. SEMs emphasize the diverse influences on dietary adherence, ranging from individual factors (such as socio‐demographic, socio‐cognitive and behavioural factors) to broader societal factors. Such broader factors include large‐scale influences, often referred to as macro‐environmental factors, such as economic conditions, food systems, cultural norms and public policies [8]. Understanding the resilience of the MD through the lens of SEM interactions offers a valuable framework for designing interventions. Research, based mainly on quantitative studies in European Mediterranean countries, shows that socio‐demographic factors such as age and gender are important, since older adults and females tend to adhere more closely to the MD than younger individuals and males [9]. In addition, higher educational attainment [10] and higher socio‐economic status [11] are associated with better adherence in European populations. Psychological factors such as positive health beliefs towards healthy eating and strong convictions about the benefits of the MD have been found to be determinants of MD adherence in several studies conducted in European Mediterranean countries [12, 13]. For instance, research conducted in Spain, Italy and Greece, predominantly based on quantitative analyses, has highlighted the importance of these psychological factors in affecting the adherence to the MD [12]. These studies underscore the role of individual beliefs and attitudes in influencing dietary choices and maintaining healthy eating patterns [13]. However, since there is a lack of evidence on determinants of MD adherence in Mediterranean countries in general, and more specifically in Lebanon, there is a need for investigating these determinants in depth. An in‐depth exploration of determinants in a new region should begin with qualitative research to explore these psychological determinants in greater depth, particularly, where specific cultural and social contexts may impact adherence to the MD [14]. Evidence from national surveys in Lebanon specifically, highlights generational differences, with younger populations showing lower MD adherence scores compared to older adults [14]. Urbanization and changing lifestyles have also contributed to this shift, as convenience‐oriented, Westernized diets have increasingly replaced traditional meals. This decline in adherence in Lebanon is consistent with trends observed in other Mediterranean countries [3] but is particularly pronounced in Lebanon due to unique sociopolitical and economic factors. Amidst the web of factors influencing dietary choices, Lebanon finds itself navigating a unique set of challenges [15]. Since 2019, the country has been reeling from its worst EC ever [15], further exacerbating existing socio‐economic disparities and food insecurity [16]. The impact of the EC, characterized by severe inflation, rising food prices, job insecurity, and difficulties in the import of food products [17], threatens the adherence to a diet known for its health benefits, potentially leading to a decline in nutritional standards and increased reliance on less healthy, processed foods [18]. A previous quantitative study demonstrated that during the EC, Lebanese population continued to prioritize foods that are culturally significant and traditional to their diet despite financial constraints [4]. The significance of environmental‐level factors, as highlighted by SEMs, plays a crucial role in shaping behaviours and outcomes. These factors include the availability and accessibility of MD components [7], and the influence of cultural norms and social support [19] and they require further investigation, particularly through qualitative methods to understand the nuanced interactions within different contexts. Additionally, there is a notable deficiency in research examining the determinants of adherence to the MD in Mediterranean countries specifically north African and Middle Eastern Mediterranean countries, with a particular paucity of studies in Lebanon.

Existing literature, based on the findings from a previous systematic review concerning barriers and enablers regarding the adherence to the MD, primarily focuses on qualitative analyses of determinants of adherence to the MD in non‐Mediterranean countries [20]. To understand the determinants of MD adherence in Mediterranean countries such as Lebanon, there is a need for qualitative research to gain initial, in‐depth insight in these determinants. In addition, it is important not only to focus on individual level determinants, but also those that derive from other levels of the SEM, including interpersonal and environmental factors. Furthermore, it is important to investigate the potential impact of the EC as a macro‐environmental factor affecting adherence to the MD. This study's insights into the resilience of the MD amidst economic fluctuations provide critical implications for public health nutritionists and policy advocates. The aim of this study was to gain insight into the multifaceted reasons affecting adherence to the MD among Lebanese adults, guided by the SEM [8] which helps to investigate micro and macro level influences, specifically how these have been impacted by the EC.

## 2 Materials and Methods

This study employs a qualitative approach based on individual face‐to‐face interviews with a purposive sample of generally healthy adults from different regions in Lebanon. It aims to explore individual beliefs and environmental influences related to adherence to the MD among Lebanese adults during the EC. The study protocol was approved by the Notre Dame University's Institutional Review Board in Lebanon (IRB #202301). This study applied the COnsolidated criteria for REporting Qualitative research (COREQ) guidelines for designing and reporting the results.

### 2.1 Participant Recruitment Strategy

Announcements about the study were posted in various public areas across Lebanon (e.g., religious sites (churches, mosques), gyms, supermarkets) to recruit diverse participants. Social media platforms (Facebook, LinkedIn, Instagram) were also utilized to promote the study and attract interested individuals. Announcements on these platforms included contact details (email and phone number) of the main researcher (C. O.) for potential participants to express interest if they met the inclusion criteria. In addition, a snowball sampling technique was employed to recruit participants. This approach involved leveraging the professional and personal networks of the Lebanese research team members (C. O., L. A. and A. A.) to identify potential participants. The team members informed individuals within their networks about the study's objectives and inclusion criteria through information letters. These informed individuals then helped to reach out to people within their own networks who met the inclusion criteria. Interested candidates were then contacted by a member of the research team, who provided them with more information about the study's aim and procedure. Those who agreed to participate then provided their informed consent on the day of the interview. Data collection continued until data saturation was reached at a total of 25 participants after reviewing the audiotapes and discussion between both interviewers (C. O. and L. A.).

### 2.2 Selection Criteria

The study targeted individuals aged 18–75, without chronic conditions, representing the general healthy Lebanese population residing in either urban or rural areas and speaking Arabic or English. This age range was selected to capture a wide spectrum of perspectives, allowing for a comprehensive understanding of the determinants of adherence to the MD.

An overview of all participants with their socio‐demographic characteristics was maintained throughout the recruitment process. Participants were recruited using convenience sampling methods. To ensure diversity in gender, age, SES and region, ongoing monitoring of socio‐demographic profiles was conducted, and additional recruitment efforts were made to target underrepresented groups through community networks, local organizations, and outreach events in diverse geographical areas.

Exclusion criteria included people suffering from chronic illnesses, co‐morbidities, and those at high risk of nutrition‐related disorders such as inflammatory bowel diseases, cardiovascular diseases, diabetes, kidney diseases, cancer, Human Immunodeficiency Virus (HIV), or conditions affecting the ability to independently choose foods, such as documented dementia, or psychological disorders. Participants were screened for high‐risk nutrition‐related disorders through self‐reported medical history by telephone before scheduling an appointment for the interview.

### 2.3 Data Collection

The interviews were conducted between June and September 2023. Interviews were initiated based on participant availability. They were held face to face upon appointment and in a place suitable for both participant and interviewer (e.g., participant's home, workplace, university, gym, etc.) using a semi‐structured interview guide to facilitate a conversational approach and to enable the primary researcher to establish rapport with participants. Interviews lasted between 20 and 40 min. A single interview was conducted with each participant.

The interviews started with a brief questionnaire to collect background socio‐demographic information it included nine questions, inquiring about sex (male/female), age (years), education level (high school, bachelor, masters or doctoral degrees), place of residence (the nine official governorates of Lebanon), marital status (single, married, widowed, divorced or in a relationship), familial status (number of children and if living alone or with other people) and current employment status (unemployed or employed full time/part time). This was followed by assessing the level of MD adherence using the validated 14 items Mediterranean Diet Adherence Screener (MEDAS) [21]. All questionnaires were interviewer‐administered. Next, the interviewer provided a detailed explanation of the main constructs of the MD using an infographic to make sure that participants knew what was meant by the MD before conducting the interview using a semi‐structured interview guide.

Interviews were conducted by two of the Lebanese researcher team (C. O. and L. A.) in the Arabic (native) or English language, depending on the preference of the participant. The interview guide initially designed in English was translated to Arabic by a native Arabic and English bilingual researcher (C. O.). The interview guide was first piloted with five people, and these interviews were discarded from the results. The pilot study served to refine the interview guide in terms of order and clarity of the questions.

### 2.4 The 14 Items Mediterranean Diet Adherence Screener (MEDAS)

The MEDAS [21] is a brief, validated questionnaire with a specificity factor of 0.57 and sensitivity factor of 0.81. It consists of 14 items that included 12 questions about the frequency of some staple MD food intake (olive oil, vegetables, fruit, red meat, animal fats, carbonated beverages, red wine, fish/seafood, nuts, commercial food, traditional dishes [with tomato sauce, garlic, onion, etc.]) and 2 questions about the preferred fat source for cooking used and meat consumed. Each item was scored as 0 (if the answer does not meet the recommendations for the specific question) or 1 (if it does), and the final score is the sum of all items (0–14). The total MD adherence score is divided into three categories: ≤ 5 corresponded to ‘low adherence’, 6–9 referred to a ‘moderate‐to‐fair adherence’, ≥ 10 corresponded to ‘good‐to‐very good adherence’ [21].

### 2.5 Interview Guide

The selected interview questions were based on the findings from a previous systematic review concerning barriers and enablers regarding the adherence to the MD (Obeid et al., in preparation) as well as on the SEM's individual, interpersonal and environmental levels [8]. The individual‐level factors mainly included socio‐cognitive determinants; interpersonal factors included perceived influence of family, friends and colleagues on the participant's food choices. The environmental level factors mainly focused on physical and economical aspects such as food availability, affordability and accessibility. Participants were able to elaborate on the barriers and enablers of adhering to the MD within the main categories of determinants (individual, interpersonal, environmental) and on how the EC has affected these factors. The interview commenced with a series of inquiries aimed at qualitatively assessing first the adherence of the participant to the MD. Subsequent inquiries explored any changes in adherence attributable to the EC. Then the interviewer delved into the factors affecting their adherence. To evaluate the influence of personal socio‐cognitive factors on MD adherence, the interviewer probed participants regarding their attitudes, beliefs, and perceived advantages or disadvantages associated with adhering to MD amidst the EC. For example: ‘What do you see as advantages/disadvantages of you adhering to MD during this EC?’. In examining the impact of interpersonal dynamics, participants were prompted to reflect on the influence of individuals in their social circles on MD adherence, elucidating specific roles and mechanisms. For instance: ‘Do the people around you play a role in your adherence to the MD? Who? and how?’ Furthermore, considerations of environmental factors were addressed through inquiries regarding external facilitators or barriers affecting MD adherence, including both physical surroundings and broader economic conditions. For example: ‘Are there any factors in your surroundings that make it hard/help you to adhere to the MD?’ and ‘Are there any factors in the financial state in general (like inflation, unemployment rates, taxation policies, and your spending habits) that are related to your adherence to the MD? What and how?’ Detailed prompts were employed to elicit comprehensive responses from participants, ensuring thorough exploration of relevant topics. A comprehensive interview guide is provided in Supporting Information: Material S1 for reference. Interviews were audio‐recorded and transcribed in Arabic, with the transcriptions securely stored immediately, and the recordings were destroyed after transcription. Quotes presented in the article were translated from Arabic to English by a native Arabic and English bilingual researcher (C. O.). The interview guide is provided in Supporting Information: Material S1.

### 2.6 Data Analysis

Researchers (L. A. and C. O.) familiarized themselves with the digital audiotapes that were transcribed verbatim in Arabic by L. A. The transcripts were not returned to participants, nor did they provide feedback on the findings. The main investigator (C. O.) designed the basis of the coding tree based on the SEM and the interview guide in consultation with two other co‐authors (A. O. and J. G.). The guide included general themes on adherence to MD, followed by subthemes on the changes due to the EC as well as themes and subthemes on the different levels of determinants: individual, interpersonal and environmental. After reviewing the first set of recorded interviews (10 recordings), additional codes were added to the coding tree for further refinement. The framework method was used for thematic analysis of the transcripts. Two researchers (C. O. and L. A.) independently coded the same three transcripts (12% of the transcripts), using Atlas.ti (version 23), to determine inter‐rater reliability (IRR). The first round resulted in an IRR of 70%. The initial findings were discussed during meetings to resolve discrepancies. Then this process was replicated on three additional transcripts (another 12%) which resulted in 92% agreement during the second round, which was considered sufficient [22]. To further confirm the similarity, another two transcripts were coded in collaboration and the remaining were divided between both researchers.

Coding involved reading and re‐reading transcripts, and coding the content into themes and subthemes until no further codes emerged. After the open coding phase, all coded quotations were translated to English by a native English and Arabic speaking researcher (C. O.), and two other researchers (J. G. and A. O.) were consulted to refine the framework and ensure consensus on themes and subthemes. The remainder of the transcripts were then coded based on the refined coding tree. The results were analyzed and reported according to the structure of the interview guide and the coding tree: first, by reporting on the main outcome which is the level of adherence to the MD both before the EC and currently and if there were any changes in adherence; then, the different determinants affecting, in general, the adherence on the individual, interpersonal and environmental levels with a focus on the impact of EC whenever there was a change in adherence.

## 3 Results

The study comprised 25 participants, aged between 21 and 73 years, with a majority (64%) being male. The religious background included 76% Christians and 24% Muslims. Geographically, the majority resided in urban settings (64%). As for the educational level, most participants had bachelor's degrees and above (76%). Employment status varied, with 72% working full‐time, 16% part‐time, and 12% not being employed. Marital status distribution showed 60% singles, 32% married individuals, and 8% divorced participants. MEDAS scores ranged from 2 to 11 indicating varying levels of adherence to the MD, with an average of 7.28, indicating a moderate adherence. Specifically, 24% of participants scored between 2 and 4 and thus had low adherence to the MD, 32% showed a moderate adherence with a score between 5 and 7, and 44% scored between 8 and 11, thus having high adherence. Detailed information on participants is presented in Table 1.

**Table 1:** MEDAS score and socio‐demographic characteristics of participants.

Number	Age (years)	Gender	Religion	Place of living	Education level	Employment status	Marital status	MEDAS score
1	25	Male	Christian	Rural	Master	Full time	Single	2
2	29	Male	Christian	Rural	Bachelor	Full time	Single	8
3	27	Female	Christian	Rural	Master	Part time	Single	11
4	24	Male	Christian	Urban	Bachelor	Full time	Single	5
5	32	Male	Christian	Urban	Bachelor	Full time	Single	4
6	39	Male	Christian	Urban	High school	Full time	Single	10
7	34	Male	Christian	Urban	Bachelor	Full time	Married	9
8	64	Female	Muslim	Rural	Bachelor	Full time	Married	6
9	30	Male	Christian	Urban	Master	Full time	Single	7
10	49	Male	Christian	Urban	Bachelor	Full time	Divorced	2
11	27	Female	Muslim	Urban	Bachelor	Full time	Single	10
12	53	Male	Christian	Urban	Bachelor	Part time	Single	8
13	29	Female	Christian	Urban	Bachelor	Full time	Single	4
14	52	Male	Christian	Urban	Doctorate	Part time	Divorced	7
15	44	Male	Christian	Rural	Bachelor	Full time	Single	10
16	25	Male	Christian	Urban	High school	Full time	Single	9
17	27	Male	Christian	Rural	High school	Part time	Single	3
18	21	Female	Christian	Urban	Bachelor	Full time	Single	9
19	68	Female	Muslim	Urban	Bachelor	Not employed	Married	10
20	53	Female	Muslim	Urban	High school	Not employed	Married	10
21	63	Female	Christian	Rural	Bachelor	Part time	Married	4
22	63	Female	Muslim	Urban	Middle school	Full time	Married	5
23	65	Female	Christian	Rural	Middle school	Not employed	Married	9
24	57	Female	Christian	Urban	Bachelor	Not employed	Married	10
25	73	Female	Christian	Urban	Middle school	Not employed	Married	10

### 3.1 Eating Habits and MD in the EC

The EC frequently emerged in interviews; this section highlights explicit changes participants reported in their dietary habits due to the EC. In the interviews, nearly all participants indicated to either completely or mostly adhere to the MD. A few indicated to not fully follow the fish recommendations. When discussing changes prompted by the EC, participants were not very explicit in how it affected their adherence to the MD. Some participants reported positive changes (P20: ‘My diet was worse before [the EC]…’), such as adopting more balanced and nutritious meals, reducing meat consumption, and increasing homemade cooking, or embraced innovation in traditional recipes, introducing new ingredients or variations to staple dishes (P8: ‘…[due to the EC] we started inventing new dishes…’). Others maintained their usual food preferences despite economic challenges. Additionally, there was a heightened awareness of food waste among certain respondents, leading to a more mindful approach to meal preparation (P25: ‘…the EC taught us to appreciate things more…’). None of the participants mentioned to have changed to poorer or more unhealthy diets.

## 4 Personal Determinants of Adherence

### 4.1 Knowledge

#### 4.1.1 General Knowledge About Nutrition

Almost all participants expressed a broad interest in food‐related topics and demonstrated knowledge of the components and health benefits of the MD. Vegetables, chickpeas, whole grain products and traditional Lebanese dishes like mutabal and hummus (traditional Lebanese dishes made of eggplants and chickpeas and sesame paste, respectively) were specifically mentioned as components of the MD. Additionally, participants also expressed good knowledge and highlighted their appreciation for the nutrition value of the MD. The emphasis on the nutritional benefits of certain foods, such as the protein‐rich hummus, further underscored their understanding of basic Mediterranean dietary principles.

#### 4.1.2 Reading Food Labels

There was a reluctance among some participants to read food labels, despite recognizing their importance. Various reasons were reported, including a lack of understanding and patience, mistrust in label accuracy (P7: ‘I don't read food labels because I don't understand them and don't trust them, especially in Lebanon…’), time constraints during supermarket visits and challenges related to small font sizes (P5: ‘the nutrition facts are small and hurt the eyes, so I get lost’).

### 4.2 Attitude/Beliefs

#### 4.2.1 Taste

Some participants expressed a commitment to consuming foods they genuinely enjoy, regardless of their origin or price (P1: ‘I don't look at the food prices because I like to buy the food I love to eat’). Some participants experimented with new food items, but only if they aligned with individual tastes, with a quick return to familiar options if the new items did not meet their expectations. This crisis‐induced change has also impacted taste preferences for a few participants, with a reduction in dining out and a shift towards more economical yet satisfying homemade options. However, some participants developed unhealthy coping mechanisms to dealing with the crisis, such as turning to comfort foods (P13: ‘… I started eating a lot of sweets, it's my comfort food’).

#### 4.2.2 Healthiness and Quality

Many participants mentioned food safety as an important reason for food choices (P14: ‘the safety of the food is very compromised, everything is expired… I choose food based on food safety mainly’) and for avoiding certain categories of foods (P25: ‘I'm scared to try any type of meat or canned products’). Health concerns were also mentioned as drivers of choices for most participants, balancing personal and family well‐being against rising medical costs (P22: ‘… the cost of medical services in Lebanon became much more expensive than the inflation in food prices’). Homemade meals were favoured for taste and health benefits by most. For almost all participants, quality was paramount in food choices, with a preference for familiar brands despite cost considerations (P18: ‘…I like to buy the brands I'm used to, the original brands’). For some, experimentation was limited to products meeting quality standards (P12: ‘I tried local food once and I didn't like the quality, so I don't buy local food anymore’). As the crisis further evolved, there was a noted transition away from price‐driven decisions towards a more stable outlook among many participants, where price considerations no longer dominated food choices as much as during the beginning of the EC (P7: ‘in the beginning of the crisis, food prices were the main drivers of my choices but now things are better…’).

#### 4.2.3 Lack of Trust

The prevailing sentiment among almost all participants was one of scepticism and uncertainty, with individuals expressing doubts about the food products available in the market (P6: ‘We live in a country that is all a lie. The food industry is more oriented towards monetary gain than quality’). Amidst these apprehensions, there was heightened focus on discerning the true quality of food items (P6: ‘We're not sure about the quality of the food anymore, if it's good or not’). Many participants highlighted concerns about low food quality due to economic constraints, which have led to prioritization of trusted, albeit more expensive, products to safeguard personal and family health.

### 4.3 Perceived Behavioural Control

Participants did not bring up lack of perceived behavioural control as a barrier for adherence to the MD. Some participants indicated a perceived ability to maintain dietary discipline within a structured routine (P4: ‘…my diet is organized during weekdays because my life is organized’). A few others referred to a perceived lack of patience for food preparation tasks as a barrier for healthy choices (P5: ‘… I don't like and don't have the patience to wash vegetables, so I go for fast foods’). Similarly, some participants suggested a reliance on others to cook traditional Lebanese dishes due to personal impatience (P22: ‘I like to eat traditional Lebanese dishes only if someone cooks them for me, because I myself don't have the patience to do so’).

### 4.4 Perceived Social Norms

Participants came up with several aspects that relate to social norms or social influences, and the need for social validation before trying new foods (P9: ‘…For me to try new things, they should be tested by many people, and I hear great feedback before I go and try it’). Cultural attitudes, such as Lebanese reluctance towards unfamiliar foods (P20), and the influence of observing others eating healthily (P18), shaped trust and acceptance of new foods for some. Familial and societal expectations overrode personal preferences for some (P6: ‘…I always eat my mum's cooked dishes even if I don't like them, I still eat them’). Economic constraints affected how some participants talked about their finances, with evolving societal attitudes towards discussing financial limitations (P6: ‘Nowadays when we can't afford some items, we're not shy to say it however before we used to be ashamed to say we can't afford something’).

### 4.5 Financial Status

Some individuals mentioned proactive financial measures taken prior to the crisis, such as moving money abroad or having income sources from other countries, which shielded them from its economic impact (P11: ‘I [my food choices] wasn't affected financially with the crisis because I take my salary from Australia’). Few others noted positive effects stemming from adjustments in spending habits necessitated by the crisis, such as refraining from fast food consumption due to affordability concerns (P2: ‘…before the crisis, I always ate fast food, now not anymore because it's not affordable, so the crisis impacted me positively’).

## 5 Interpersonal Determinants

### 5.1 Family Influence

For many participants, families significantly influenced dietary habits, with parents directly guiding healthy eating practices (P8: ‘I taught my children from a young age to read food labels’). Eating together and relying on home‐cooked meals further underscored this influence. Seeking advice from family members also shaped food behaviours. For a lot of participants, family norms often override other social influences (P17: ‘Even if my colleagues eat different food items, I bring my mum's home‐cooked food for lunch at work’). Individuals living away from their families were influenced by occasional interactions or got food from family members (P2: ‘Sometimes my mum sends me food to my dorms’). Family composition impacted dietary patterns, with those living alone often resorting to convenience foods due to a lack of someone to cook for them. For few, changes like divorce or the death of the partner also altered dietary choices. Adapting to a partner's preferences was common, with some prioritizing their partner's taste over their own. Smaller households bought fewer perishables to minimize waste, potentially reducing financial strain.

### 5.2 Friends' and Colleagues' Influence

Interviews revealed a general resistance to social influences, with some prioritizing research and experience, while a few others admitting indirect influence (P17: ‘…sometimes I observe whether people around me have tried some diets or products…’). Some participants mentioned the influence of friends and colleagues on their dietary choices. For example, the communal nature of Lebanese food discouraged ordering it alone (P1: ‘… I don't order Lebanese food because we should be with many to eat it, the Lebanese mezze is made to be shared’). Roommate's and friends' advice and habits impacted food choices for some (P12: ‘I'm influenced by what my friends at the gym tell me about food’). Very few participants cited colleagues' influence on food choices, accepting offerings despite personal preferences. Conversely, more participants asserted independence, (P11: ‘my colleagues don't influence me’).

### 5.3 Social Media Influence

Some younger participants highlighted social media's impact on diet and lifestyle choices. For instance, exposure to others' successes with certain diets or foods on platforms like Instagram often prompted emulation. Influencers played a significant role in guiding food and diet choices for some (P17: ‘everything I see on social media is important to me’). Additionally, younger participants prioritized social media appearances over traditional mealtime rituals, like setting tables for aesthetic photos rather than for eating.

## 6 Environmental Factors

### 6.1 Food Affordability

The majority of participants noted changes in food affordability due to economic challenges (P19: ‘… there are a lot of products that I like but I stopped buying because they got more expensive’) reflecting a shift away from preferred items, (P6: ‘… nowadays I have to do my calculations [of which products I can buy]…’) indicating a need to adapt to increased expenses. Some altered food choices based on price considerations, (P7: ‘nowadays we cannot afford expensive food like meat and poultry… relying more on cheap options like pasta and rice’). However, some prioritized preferences over price constraints (P10: ‘I buy the food products I want regardless of their price’). Adaptations were observed among many overtime (P25: ‘… now we got used to it [the EC]… What matters to me is… good quality’), which highlighted the need for participants to navigate food affordability challenges and adjust consumption habits in response to economic fluctuations.

### 6.2 Food Availability

Almost all participants noted shortages of familiar items (P20: ‘… we couldn't find the products we are used to, so we got lost’). Some took proactive measures, like buying freezers for storage. This situation positively influenced eating habits for some participants, fostering adherence to the MD due to the availability of local food (P21). However, concerns about local product quality led some to a preference for imported items (P14: ‘I buy imported food items because I trust their quality’). Challenges in finding preferred products prompted shifts in consumption behaviour for many. Safety concerns arose for most participants, with worries about expired products and compromised standards (P5: ‘demand has decreased so a lot of food products weren't sold and remained in the market near to their expiry dates and weren't stored in the right conditions’).

The place of residence seemed to influence participants' lifestyle choices and availability of resources. For instance, some of the participants residing in smaller towns or villages faced limitations in terms of shopping options and product variety, as illustrated by (P23: ‘We live in a small town, the shops have a very limited variety and are not of good quality therefore, I do my big shopping from a big supermarket [in a larger town]’). Conversely, those from rural areas benefited from proximity to homegrown produce and livestock, as exemplified by (P1: ‘I'm from a village. We grow fruits and vegetables next to the house; my grandpa has chickens and can get eggs’) indicating a reliance on locally sourced foods. Moreover, the sense of attachment to one's hometown or village lead, for some, to a stronger adherence to traditional dietary practices, as seen in the statement of (P1: ‘When I'm in my hometown [town in Lebanon], I adhere more [to the MD]’).

### 6.3 Food Accessibility

Most participants prioritized convenience and proximity over cost in their purchasing decisions (P13: ‘I buy more from the grocery shops next to my house… they are convenient’). Delivery services for grocery shopping demonstrated efforts to ensure access to essentials while minimizing travel. Local grocery stores offered for few participants personalized services, such as sending pictures for selection (P22: ‘I order food items from the grocery shop next to my home… he sends me pictures… I choose and he delivers’).

## 7 Discussion

This study aimed to explore factors influencing adherence to the MD through the lens of the SEM, particularly focusing on how the EC in Lebanon has affected this. Based on the quantitative assessment, results indicated a wide range in adherence to the MD, with MEDAS scores spanning from 2 to 11. Although the average of MEDAS showed moderate adherence (average score 7.28), this does not reflect the distribution accurately. In fact, 44% of participants achieved scores between 8 and 11, indicating high adherence to the diet. However, these figures cannot be interpreted as an indication of average adherence to the MD in Lebanon, given the small sample size of the current study. Moreover, in the interviews, participants expressed a belief in better adherence to the MD than indicated by their MEDAS scores, which indicates a lack of awareness of their own behaviour among some participants. This is often seen in complex health‐related behaviours such as dietary behaviours [23], and this lack of awareness may, in turn, limit the motivation to change [24].

The results showed that during the EC, most participants seemed to improve or maintain their eating habits, as reflected in their descriptions of increased home cooking, greater use of fresh ingredients, and a stronger focus on balanced meals. While a few participants mentioned consuming more comfort foods as a coping mechanism, none explicitly indicated adopting an overall unhealthier diet. These findings are in contrast to those found in other studies where economic hardships led to unhealthier food choices, because these were the cheapest [25]. While participants indeed turned to the cheaper options in the current study, cheaper options are, in the Lebanese context, the healthier options, such as more traditional food and home cooking. These types of food and cooking align with the MD principles, mainly based on legumes with minimal use of red meat and processed food. The shift towards homemade meals and reduced dining out as cost‐saving measures is a common response during economic crises, highlighting a preference for cost‐efficiency and cost control [26]. The finding that people improved or maintained their diet during the crisis is in line with a previous study in Lebanon that showed that during the Lebanese EC people have been turning to more affordable food options, which often include healthier choices such as vegetables and legumes [4]. This previous study also showed that many people continued to prioritize foods that are culturally significant and traditional to their diet, reflecting the strong cultural ties to food and eating habits in the region [4]. Moreover, the United Nations report on the economic and COVID‐19 crises in Lebanon notes that the crisis has forced many citizens to adopt more economical lifestyles, inadvertently leading to healthier eating habits. This shift is primarily because highly processed and imported foods have become too expensive for the average consumer, driving them towards locally produced, healthier alternatives [27]. All in all, the effects of the crisis appeared to be generally not unfavourable in regards of the participant's diet. An additional explanation for findings not in alignment with those of previous studies can be first due to the fact that during the EC, some continued to earn their salaries in foreign currencies while others were paid the deflating Lebanese currency which led to a segregation in the social classes [15]. Also, it is important to note that the EC in Lebanon coincided with the COVID 19 pandemic, which could have made people more alert to their health, in a country where health insurance became too expensive, as it was noted with previous studies assessing the impact of COVID 19 on general eating behaviours [28, 29].

On the personal level, the findings indicated a lack of trust and pinpointed quality as an important factor towards food choices during the EC, reflecting broader patterns observed in the literature [30]. The finding that scepticisms and distrust played a role is in line with findings of previous studies in which economic downturns often lead to widespread scepticism about food quality, with concerns about cost‐cutting measures reducing food standards [26, 27, 28]. This trend is evident in the current participants from Lebanon, where many prioritize trusted brands despite higher costs to ensure safety and quality.

On the interpersonal level, the influence of family, friends and colleagues on dietary habits was evident but varied in intensity. Family influences were generally profound, with parents instilling healthy eating practices and family norms, often overriding other social influences. For individuals living alone, family interactions and food sent from home continued to shape their dietary behaviours. This is not unusual in Lebanese culture, where the influence of family ties and pressure is profoundly ingrained and often surpasses the impact of peer pressure, even among older age groups [31]. Family influence on diet is well‐documented, particularly in promoting healthy eating habits from a young age [32]. Parents shape their children's dietary behaviours through the foods they provide, meal routines, and their own eating habits, which children tend to emulate. This early exposure to healthy foods and positive eating patterns helps establish long‐term dietary preferences and habits [33].

Friends' influence on dietary choices is notable through social norms and communal eating habits, such as positive encouragement from friends. Social networks, including friends and colleagues, shape dietary behaviours through these norms and indirect influences [34]. Colleagues' influence was found to be minimal, with some asserting independence from workplace norms, though a few acknowledged the indirect impact of observing colleagues' dietary habits. Social media significantly affected younger participants' dietary choices, reflecting broader modern food consumption trends where social media platforms shape eating behaviours by promoting visually appealing and trendy foods [35]. Studies indicate that young people are highly susceptible to food marketing on social media, impacting their food preferences and consumption patterns [36]. Leveraging social media to promote healthier eating can be highly effective but it is crucial to incorporate critical media literacy to help young adults make informed food choices [36]. Integrating educational components into health promotion strategies can enhance adherence to healthy diets, such as the MD, by teaching critical evaluation of food content on social media [36].

The economic challenges significantly influenced the participants' food environment, particularly in terms of affordability, availability and accessibility of certain foods. Many participants reported a noticeable increase in food prices, which compelled them to shift away from their preferred items to more affordable alternatives. This shift is consistent with existing literature, which indicates that economic downturns often lead to reduced expenditure on higher‐cost food items like meat and poultry, resulting in a dietary shift towards cheaper staples [37]. Nonetheless, a minority of participants continued to prioritize their food preferences over price constraints, highlighting a variability in adaptation strategies. Understanding the diverse adaptation strategies among individuals can help tailor support programmes to meet varying needs and preferences, ensuring more effective dietary interventions during economic downturns [38].

In terms of food availability, participants experienced significant disruptions, with shortages of familiar items causing confusion and prompting proactive measures such as storing food at home. This aligns with findings in the literature that suggest economic instability often leads to food supply issues, influencing consumer behaviour towards stockpiling and altering dietary patterns [39]. Interestingly, some participants reported a positive shift towards the MD due to increased reliance on locally available foods despite concerns about the quality of these local products. This nuanced response underscores the complex interplay between availability, perceived quality, and dietary choices during economic crises [40].

There seemed to be a difference in dietary choices according to the place of residence in the current study, as was also evidenced by a study on elderly populations in the Mediterranean islands [41] that found that rural residents generally have better adherence to traditional dietary practices largely due to the availability and accessibility of fresh produce and traditional foods [41]. Regarding food accessibility, convenience and proximity emerged as critical factors in purchasing decisions for most participants, who preferred local grocery shops and valued services like home delivery and personalized shopping assistance. This preference for convenience over cost is supported by existing research which suggests that accessibility and ease of procurement become paramount when consumers face economic and logistical challenges [42].

This study provides in‐depth insights into personal, social, and economic factors influencing dietary behaviours, revealing how financial constraints affect food choices and diet quality. The inclusion of 25 participants with diverse demographic characteristics ensures a broad range of perspectives, strengthening the depth and richness of the findings. While the purpose of this qualitative exploration is not to produce generalizable results, the study provides valuable insights into the determinants of adherence to the MD. The findings contribute to a deeper understanding of behavioural patterns and influencing factors, which can inform future research, policy development, and targeted nutritional interventions. Data saturation was reached, reinforcing the credibility and robustness of the themes identified. The findings also underscore the intricate relationship between food culture and food systems, illustrating how community traditions and policy‐level supports sustain dietary patterns despite economic pressures. This interplay aligns with systems thinking, emphasizing the dynamic interdependence of SEM levels in shaping resilient food practices. Nonetheless, further subgroup differences possibly exist, for instance, between older and younger participants, as appeared to be the case for some of the identified themes. Future research should further examine such subgroup differences. The qualitative approach allows us to capture more understanding of the lived experiences and perceptions of the participants that quantitative methods might miss.

However, there are limitations to consider. As a broad range of topics was discussed, not all aspects were elaborately covered in depth. Moreover, participants may answer in a socially desirable way as well as the interviewer skills could have influenced the depth of discussion. Also, almost all participants expressed a broad interest in food‐related topics which could be pointing out that we have recruited a selective sample. However, it was diverse in terms of age, gender, education, marital status, etc. Therefore, for the aim of this study, to generate initial in‐depth insight into potential determinants of MD adherence during the economic crisis, this sample was sufficient. Nevertheless, it would be recommendable to repeat this study among a population with different characteristics. The broad age range also could be a limitation, and the sample was not sufficiently large to make distinctions based on age group or other characteristics. Therefore, future studies could focus on getting deeper insight in subgroups divided by age or other socio‐demographic characteristics. Additionally, all qualitative studies can be subject to researcher bias in data interpretation. However, the data were analyzed by two independent researchers, showing an IRR of 92%, which is high [22]. Despite the limitations, the detailed qualitative insights can inform public health strategies and policymaking, particularly in addressing economic barriers to healthy eating and promoting adherence to the MD during economic downturns.

## 8 Conclusion

This study explored individual, interpersonal and environmental determinants of adherence to the MD during Lebanon's EC. Quantitative assessment showed moderate adherence, while qualitative interviews suggested participants believed they adhered well, indicating a lack of awareness of their actual behaviours. Key determinants included individual attitudes beliefs, specifically importance of food quality and lack of trust in food products in the Lebanese market, and significant environmental factors like food availability, accessibility and affordability, with social influences playing a minor role. Despite economic constraints, many participants prioritized diet quality, driven by their attitudes and beliefs. Future research should focus on further investigating the determinants of MD adherence, to be able to promote MD adherence that was declining in Lebanon. Additionally, people shifted to more traditional and healthy diets due to financial reasons and increased appreciation for traditions. Emphasizing the importance of tradition could be a key focus for interventions.

## Author Contributions

Cécile Obeid, Anke Oenema and Jessica S. Gubbels conceptualized the study. Cécile Obeid conducted the literature search. Cécile Obeid, Anke Oenema and Jessica S. Gubbels designed the interview guide. Cécile Obeid, Luna Awad and Antoine Aoun recruited participants. Cécile Obeid and Luna Awad conducted the interviews. Luna Awad transcribed verbatim the digital audiotapes to Arabic. Cécile Obeid designed the basis of the coding tree based on the SEM and the interview guide in consultation with two other co‐authors Anke Oenema and Jessica S. Gubbels. Cécile Obeid and Luna Awad independently coded the transcripts. Cécile Obeid translated all coded quotations to English. Cécile Obeid wrote the original draft of the manuscript. Cécile Obeid, Anke Oenema, Jessica S. Gubbels, Antoine Aoun and Stef P.J. Kremers critically revised and edited the manuscript. All authors have read and approved the final version of the manuscript.

## Ethics Statement

This study was conducted according to the guidelines laid down in the Declaration of Helsinki and all procedures involving research study participants were approved by the Notre Dame University's Institutional Review Board in Lebanon (IRB #202301).

## Consent

Written informed consent was obtained from all subjects/patients.

## Conflicts of Interest

The authors declare no conflicts of interest.

### 1 Peer Review

The peer review history for this article is available at https://www.webofscience.com/api/gateway/wos/peer-review/10.1111/jhn.70053.

## Supporting information

Supporting Material S1 1.

## Data Availability

The data that support the findings of this study are available from the corresponding author upon reasonable request.
