# Supplementary material for: Exploring Determinants of Adherence to the Mediterranean Diet Among Adults in Lebanon During the Economic Crisis: A Qualitative Study
Source: J Hum Nutr Diet. 2025 Apr 15;38(2):e70053. doi: 10.1111/jhn.70053 (PMC11997636; doi:10.1111/jhn.70053)
Supplement: Supplementary file 1 — Supporting Material S1 1. [file JHN-38-0-s001.docx]

**Legend for Supplementary Material S1:**
Supplementary Material S1: Interview guide designed based on the Socioecological Model (SEM) to assess individual, interpersonal, and environmental determinants influencing adherence to the Mediterranean Diet.

| **Main themes** | **Main question** | **Follow-up questions** | **Prompts/check for determinants** |
| --- | --- | --- | --- |
|  |  |  |  |
| Opening/ice-breaker | What is your favorite food? |  |  |
| What is MD | What do you think the MD is? How would you describe the MD? |  |  |
| **The interviewer explains the main constructs of the MD; use an illustration/ picture of the MD** | | | |
| Current behavior: Compliance with MD and elements | What do you comply with and what don’t you comply with within the MD? | why do you comply/not comply to these elements of the MD? |  |
| Changes due to EC | Has it always been like that or has it changed?  Did you intend to change? | How has it changed?  Was this change related to the EC? |  |
| Most important reasons/causes for that change | What were the most important reasons for this change (be it positive or negative) during this EC?  In case of no change what enabled you to keep on following a Mediterranean-style diet during this EC?  In case of no adherence and no change, can you explain the reasons behind your non adherence? | Can you talk more about that?  Were there other important reasons? |  |
|  | You have been talking about…. Are there any other things that determine your level of adherence to the MD?  *You can think of your personal reasons, thoughts, opinions or other things.*  *Or people and things that are around you.* |  |  |
| Factors at the intrapersonal level | Personal reasons and opinions can also play a role. Can you think about that…? | Can you explain, how (*name the prompts if not tackled by the participant*) affect your adherence to MD? | Socio economic: (*Income*  *Education*  *Occupation*  *Employment*  *Marital status)*  Food habits: how does following a MD impact your everyday eating habits?”  Tradition  Religion  Beliefs and Attitudes: What do you see as the advantages of you adhering to MD during this EC?’, ‘What do you see as the disadvantages of you adhering to MD during this EC?’ and ‘What else comes to mind when you think about adhering to MD during this EC?’  Skills: are you able to cook a meal according to MD? Do you know which food to select according to MD? skills to read package labels  Self-efficacy: "How confident are you in your ability to choose/preparing/eating food according to MD?"  Risk perception Can you describe your personal ideas of the risk associated with not adhering to MD?  personal norm: Sometimes, when we are not sure what to do, we look to see what others are doing. Please list the individuals or groups you think are most likely to adhere to MD’; and ‘Please list the individuals or groups who are least likely to adhere to MD’. |
| Factors at the interpersonal level/social environment | Do the people around you play a role in your adherence to the MD?  Who? How does that work? | Can you explain, how the influence of (*name the prompts if not tackled by the participant*) affect your adherence to MD?  What kind of influence do you get form (*name the prompts if not tackled by the participant*)? | Family  Friends  Work (colleagues, boss)  Community  Practical/informational/emotional… help  Social support  Social pressure |
| Factors at the physical environmental level | Are there any factors in your surroundings that are related to your adherence to the MD?  *Note to self: if people don’t respond use the below questions:*  Are there things in your surroundings that make it hard for you to adhere to the MD?  Are there things in your surroundings that help you adhere to the MD? | Can you explain, how (*name the prompts if not tackled by the participant*) affect your adherence to MD? | Household  Neighborhood  Food retails  Restaurants  Media/ Advertisement  Food labels  Food safety  Food quality (good quality food items less available OR relying on locally grown food items)  Food availability  Food accessibility |
| Factors at the economic environmental level | Are there any factors in the financial state in general (like inflation, unemployment rates, taxation policies and your spending habits) that are related to your adherence to the MD?  *Note to self: if people don’t respond use the below questions:*  What in the financial state in general (like inflation, unemployment rates, taxation policies and your spending habits) make it difficult for you to adhere to the MD?  What in the financial state in general (like inflation, unemployment rates, taxation policies and your spending habits) help you adhere to the MD? | Can you explain, how (*name the prompts if not tackled by the participant*) affect your adherence to MD? | Food affordability  Food prices  (of items that fit within MD and items that do not fit with MD) |
| COVID related factors |  |  |  |
